# Occupancy-Aware Neural Distance Perception for Manipulator Obstacle Avoidance in the Tokamak Vacuum Vessel

**DOI:** 10.3390/s26010194

**Published:** 2025-12-27

**Authors:** Fei Li, Wusheng Chou

**Affiliations:** School of Mechanical Engineering & Automation, Beihang University, Xueyuan Road 37, Beijing 100191, China; 695725811@buaa.edu.cn

**Keywords:** neural distance perception, occupancy-aware sampling, tokamak vacuum vessel, neural signed distance fields, real-time collision checking

## Abstract

Accurate distance perception and collision reasoning are crucial for robotic manipulation in the confined interior of tokamak vacuum vessels. Traditional mesh- or voxel-based methods suffer from discretization artifacts, discontinuities, and heavy memory requirements, making them unsuitable for continuous geometric reasoning and optimization-based planning. This paper presents an Occupancy-Aware Neural Distance Perception (ONDP) framework that serves as a compact and differentiable geometric sensor for manipulator obstacle avoidance in reactor-like environments. To address the inadequacy of conventional sampling methods in such constrained environments, we introduce a Physically-Stratified Sampling strategy. This approach moves beyond heuristic adaptation to explicitly dictate data distribution based on specific engineering constraints. By injecting weighted quotas into critical safety buffers and enforcing symmetric boundary constraints, we ensure robust gradient learning in high-risk regions. A lightweight neural network is trained directly in physical units (millimeters) using a mean absolute error loss, ensuring strict adherence to engineering tolerances. The resulting model achieves approximately 2–3 mm near-surface accuracy and supports high-frequency distance and normal queries for real-time perception, monitoring, and motion planning. Experiments on a tokamak vessel model demonstrate that ONDP provides continuous, sub-centimeter geometric fidelity. Crucially, benchmark results confirm that the proposed method achieves a query frequency exceeding 15 kHz for large-scale batches, representing a 5911× speed-up over mesh-based queries. This breakthrough performance enables its seamless integration with trajectory optimization and model-predictive control frameworks for confined-space robotic manipulation.

## 1. Introduction

The tokamak is a magnetic confinement device designed to harness energy from controlled nuclear fusion. The Vacuum Vessel (VV) constitutes the core component of the tokamak, serving as the direct operating environment for the plasma [1,2]. During operation, in-vessel components are exposed to extreme thermal loads, mechanical stress, and neutron irradiation. To ensure the long-term safety and stability of the tokamak device, regular inspection and maintenance are essential.

These tasks are typically performed by heavy-duty, long-reach hyper-redundant manipulators operating in confined, complex environments [3,4]. As highlighted in recent reviews on remote maintenance [5], the primary challenge lies in navigating these high-DoF robots through small-scale access ports to reach disparate in-vessel components.

However, during maintenance, the gamma radiation dose rates inside the vacuum vessel can reach up to 2000 Sv/h [6,7]. Under such conditions, common sensors (e.g., cameras, LiDARs) suffer from signal degradation or failure. Consequently, path planning reliant on external perception is infeasible. Instead, robotic operations must shift to offline motion planning, where trajectories are pre-generated based on a prior geometric model and executed in an open-loop manner.

The reliability of such offline planning hinges fundamentally on rigorous collision checking. This is particularly critical given the extreme spatial constraints. For instance, maintenance planning for the DTT tokamak [8] necessitates strict obstacle avoidance for the HyRMan robot within the vacuum vessel, where the clearance between the manipulator and the vessel inner walls is strictly limited [6]. Furthermore, unlike rigid industrial robots, these heavy-duty long-reach manipulators exhibit significant elastic deformation under load. This physical uncertainty reduces the effective safety margin, implying that the collision checking module must provide millimeter-level accuracy to prevent catastrophic damage to internal components.

Existing collision checking algorithms, however, struggle to meet these requirements. Methods based on discrete representations, such as voxel grids, OctoMap [9], or truncated signed distance fields (TSDF) [10], suffer from a trade-off between resolution and memory consumption, often resulting in aliased (“staircase”) surfaces. On the other hand, mesh-based collision libraries like FCL [11] provide exact results but incur high computational costs when querying high-fidelity reactor models. Crucially, these discrete or mesh-based methods are typically non-differentiable, preventing the use of gradient-based optimization for generating smooth, safe trajectories in narrow corridors.

Neural implicit representations, particularly Neural Signed Distance Fields (Neural SDFs), offer a promising alternative [12,13]. By mapping coordinates continuously to signed distances, Neural SDFs theoretically offer infinite resolution and analytical gradients. Recent advancements have successfully applied Neural SDFs to mobile [14] and aerial robotics [15], demonstrating the benefits of differentiable fields for trajectory optimization. However, these approaches often prioritize online update speeds for local replanning [16] or focus on visual fidelity [17], sometimes compromising global metric accuracy through approximations. Other industrial approaches rely on decomposing space into explicit convex corridors [18], which is computationally intractable in the highly non-convex topology of a tokamak.

Applying standard Neural SDFs to fusion environments remains challenging due to specific industrial constraints: (i) heuristic sampling distributions that fail to account for mechanical elasticity and engineering safety factors, (ii) the difficulty of enforcing strict millimeter-level engineering tolerances within abstract normalized spaces, and (iii) the need for absolute offline safety guarantees rather than online approximations.

In this work, we address these limitations by proposing an Occupancy-Aware Neural Distance Perception (ONDP) framework. This model learns a continuous, high-fidelity signed distance field specifically tailored for offline motion planning in tokamak environments.

The proposed ONDP pipeline integrates occupancy-aware filtering with a Physically-Stratified Sampling strategy guided by Finite Element Analysis (FEA). By incorporating deformation data directly into the sampling logic, we achieve 2–3 mm precision where it matters most. We train our network directly in physical metric units (consistent with high-precision metrology [19]) to ensure geometric consistency across the reactor scale. The resulting model supports efficient, differentiable distance queries, enabling planners to generate safe, boundary-constrained trajectories through the narrowest reactor ports.

### Contributions

The main contributions of this work are:

- A novel Occupancy-Aware Neural Distance Perception (ONDP) framework specifically tailored for tokamak vacuum vessels. Targeting this large-scale complex scenario, we propose, for the first time, a high-precision distance prediction method capable of providing continuous, sensor-less geometric representations. This approach addresses the limitations of low accuracy and high latency in traditional collision detection methods, laying a solid foundation for high-precision and efficient motion planning in such environments.
- A Physically-Stratified Sampling strategy derived from Finite Element Analysis (FEA). Unlike heuristic methods, this strategy explicitly compensates for the heavy-duty manipulator’s elasticity by enforcing dense supervision in safety-critical deformation buffers, ensuring strictly bounded prediction errors.
- A continuous collision checking mechanism validated by simulation benchmarks. The proposed method achieves a query frequency exceeding 15 kHz, demonstrating a 5911× acceleration over mesh-based methods to support high-precision, real-time trajectory optimization.

## 2. Methodology

### 2.1. Overall Framework

**Figure 1:**
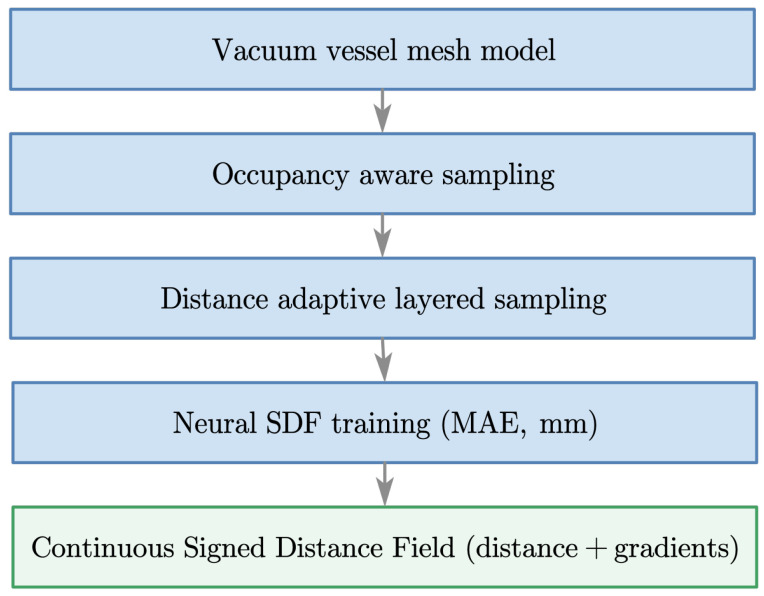
Overview of the ONDP modeling pipeline, including geometry preprocessing, occupancy-aware sampling, Physically-Stratified Sampling, and neural SDF training. The resulting continuous ONDP provides both distance and gradient information for real-time perception and planning in confined vessel environments.

Figure 1 illustrates the proposed ONDP (Occupancy-Aware Neural Distance Perception) modeling pipeline, which builds a continuous and differentiable geometric representation of the tokamak vacuum vessel. Starting from the CAD/mesh model, occupancy filtering and Physically-Stratified Sampling are used to generate geometry-aware supervision. A neural signed distance field is then trained in physical units (millimeters) using a mean absolute error loss. The resulting model provides approximately 2–3 mm distance accuracy and gradients via automatic differentiation, enabling real-time geometric queries for perception, monitoring, and motion planning in confined vessel environments.

The pipeline consists of three main components:

1. Geometry preprocessing. The vacuum-vessel mesh is validated for completeness, and surface watertightness is enforced. For correct signed-distance computation throughout the workspace, outward-facing normals are oriented away from the cavity centroid on the outer surface, while inward-facing normals are oriented toward the centroid on the inner surface. This guarantees consistent sign conventions for the distance field.
2. Occupancy-Aware and Physically-Stratified Sampling.
  Candidate samples are generated both in the cavity and within a thin in-wall shell. An occupancy test combining normal-based and winding-number checks discards invalid points.
  A Physically-Stratified Sampling strategy allocates denser samples near the wall and sparser samples toward the interior of the vessel, providing geometry-aware supervision.
3. Neural SDF learning. A multilayer perceptron maps 3D coordinates to metric signed-distance values. The model is trained using a mean absolute error loss without additional weighting or regularization, preserving metric consistency across the full workspace.

The trained ONDP supports continuous distance evaluation and gradient extraction through automatic differentiation, providing a compact, differentiable geometric field suitable for real-time collision checking and boundary-constrained trajectory optimization.

### 2.2. SDF Representation

In the proposed ONDP framework, the tokamak vacuum vessel is modeled as a continuous signed distance field (SDF) that assigns for each point $x\in R^{3}$ its signed Euclidean distance to the closest surface of the vessel interior. Let Ω denote the solid wall geometry and ∂Ω its boundary. The signed distance function is defined as

(1) $$\varphi \left(x\right)=\left\{\begin{array}{cc} -d(x,\partial \Omega ), & x\in \Omega ,\\ +d(x,\partial \Omega ), & x\notin \Omega , \end{array}\right.$$

where d(x,∂Ω) is the shortest Euclidean distance from x to the wall surface. According to a conventional robotics sign convention, negative values indicate points inside the solid wall, whereas positive values correspond to locations within the robot-accessible cavity.

The gradient ∇φ(x) provides the outward surface normal direction when x approaches ∂Ω; in practice, the normalized gradient ∇φ(x)/∥∇φ(x)∥ is used to obtain the unit normal for downstream computations such as distance-to-wall estimation and contact reasoning. Ideally, an exact signed distance field satisfies the Eikonal property ∥∇φ(x)∥=1. In practice, neural approximations slightly deviate from this condition, but ONDP preserves near-Eikonal behavior implicitly through dense metric supervision without an explicit gradient regularizer.

Compared with discrete geometric models such as voxel grids, TSDFs, or polyhedral collision meshes, a continuous SDF representation offers four key advantages in confined-space environments:

1. Continuity: enables distance evaluation at arbitrary 3D locations without voxel discretization artifacts.
2. Differentiability: provides smooth gradients yielding consistent surface normals for geometry-aware computation.
3. Compactness: encodes geometry within neural network parameters rather than dense volumetric grids.
4. Generality: represents highly non-convex, tunnel-like vessel interiors with narrow clearances.

This SDF formulation serves as the fundamental geometric representation throughout ONDP.

It guides Physically-Stratified Sampling, supervises neural training, and supports real-time inference. All computations are performed in physical units (millimeters) to maintain metric fidelity within the meter-scale tokamak cavity.

### 2.3. Physically-Stratified Sampling Strategy

To guarantee collision safety given the elasticity of heavy-duty manipulators, we propose a Physically-Stratified Sampling strategy. Instead of relying on heuristic adaptations, this approach explicitly dictates the data distribution based on Finite Element Analysis (FEA) data and engineering safety protocols.

**Figure 2:**
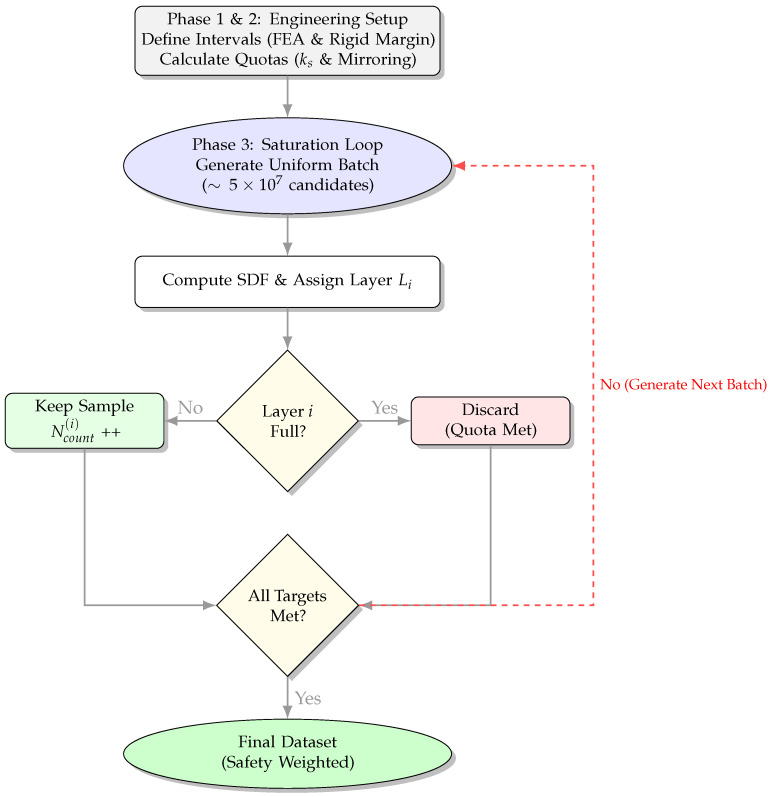
Flowchart of the sampling strategy. Phases 1 and 2 establish the FEA-derived intervals and safety quotas. Phase 3 executes the Saturation Rejection Sampling loop to strictly enforce these physically-constrained distributions.

The strategy is implemented in three logical phases, as illustrated in Figure 2:

1. Engineering-Driven Discretization

First, we partition the sampling domain into functional intervals based on the physical requirements of the maintenance task. A minimum Rigid Safety Margin of 20 mm is strictly required to prevent collision.

However, finite element simulation of the heavy-duty manipulator under load revealed a maximum end-effector deformation of approximately 30 mm. To account for this uncertainty, we extend the critical sampling boundary by the magnitude of the deformation. Consequently, the Effective Critical Region is defined as 50 mm (20+30), which is discretized into two engineering layers:

- Rigid Safety Margin (0–20 mm): This layer represents the absolute safety buffer. We strictly enforce high sampling density here to minimize prediction error. This ensures that the prediction deviation remains significantly smaller than the safety margin (ϵ≪20 mm), guaranteeing collision-free operation even under uncertainty.
- Deformation Buffer (20–50 mm): This layer covers the 30 mm potential positional drift caused by manipulator elasticity, ensuring the network perceives obstacles before they breach the rigid margin.

2. Safety-Factor Weighted Quota

Preliminary training indicated that the training error under standard sampling distributions did not meet requirements in these two critical regions. Therefore, we introduced empirical engineering safety factors ($k_{s}$) to increase the sample density where kinematic uncertainty is high.

**Figure 3:**
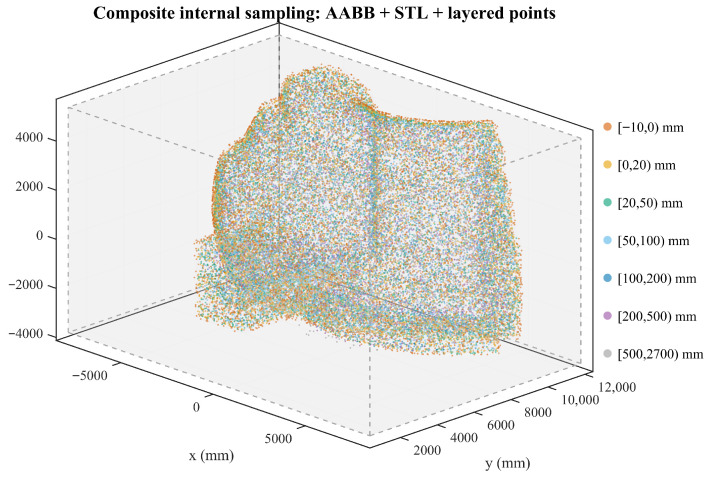
Spatial distribution of the training samples generated by the proposed strategy. Two key density peaks are explicitly enforced: (1) the deformation buffer (20–50 mm) is intensified ($k_{s}=1.5$) to counteract FEA-predicted elasticity, and (2) the interior margin (−10–0 mm) is symmetrically aligned with the rigid safety zone to ensure accurate gradient learning at the surface.

Specifically, the resulting spatial distribution is shown in Figure 3. We apply $k_{s}=1.2$ to the Rigid Safety Margin (0–20 mm) and $k_{s}=1.5$ to the deformation buffer (20–50 mm). Furthermore, for the interior boundary (−10–0 mm), we enforce a symmetric sample count to match the Rigid Safety Margin, ensuring accurate gradient learning at the surface. The detailed configuration of all sampling layers is provided in Table 1.

3. Saturation Rejection Sampling

To strictly enforce these calculated quotas, we employ a “Saturation Rejection Sampling” algorithm. Samples are generated uniformly within the expanded bounding box and retained only if they fall into a specific layer whose target quota has not yet been filled. This process iterates until all safety-weighted quotas are satisfied.

### 2.4. Neural Network and Training Objective

We adopt a feedforward Multilayer Perceptron (MLP) $f_{\theta }:R^{3}\;\to \;R$ to regress the signed distance at each 3D query point. Given $x=\left(x,y,z\right)\in R^{3}$ (in millimeters), the network outputs a scalar ${\hat{\varphi }}_{\theta }^{\mathrm{norm}}\left(x\right)$ representing the normalized signed distance.

**Figure 4:**
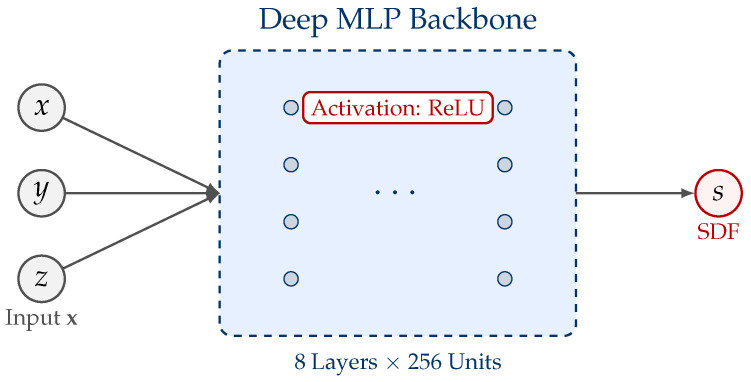
Network architecture of the proposed ONDP method. The model takes 3D coordinates (x,y,z) as input, processes them through a deep MLP backbone (8 layers, 256 units each) with ReLU activations, and outputs the continuous signed distance field (SDF) value.

The network architecture is illustrated in Figure 4. The architecture consists of an input projection layer (3→256), followed by eight fully connected hidden layers with 256 units and ReLU activation, and a final linear output layer (256→1). No skip connections or normalization layers are applied, as the target function is smooth and well-scaled in physical units.

This deep architecture ensures sufficient capacity to approximate the complex topology of the tokamak interior.

Training supervision is provided by the dataset generated via Physically-Stratified Sampling (Section 2.3), ensuring balanced coverage across the critical near-wall and far-field regions.

To ensure numerical stability during network optimization, standard min–max scaling is applied to the input coordinates and target values. However, unlike typical machine learning tasks that minimize loss in the normalized latent space, our framework strictly enforces metric consistency. The predicted normalized values ${\hat{\varphi }}_{\theta }^{\mathrm{norm}}\left(x\right)$ are mapped back to the physical scale (millimeters) using the inverse transformation:

(2) $${\hat{\varphi }}_{\theta }\left(x\right)={\hat{\varphi }}_{\theta }^{\mathrm{norm}}\left(x\right)\;\left(\varphi_{\max}-\varphi_{\min}\right)+\varphi_{\min},$$

where $\left[\varphi_{\min},\varphi_{\max}\right]=\left[-500,\;2700\right]\;\mathrm{mm}$ represents the physical bounds of the distance field. Consequently, the network optimization is driven directly by the absolute error in physical units:

(3) $$L\left(\theta \right)={∥{\hat{\varphi }}_{\theta }\left(x\right)-\varphi \left(x\right)∥}_{1}.$$

This strategy effectively decouples the numerical scaling required for gradient descent from the physical supervision required for engineering safety.

## 3. Experiments and Results

### 3.1. Implementation Details and Training Dynamics

All experiments were conducted on a workstation equipped with an NVIDIA RTX 4080 GPU (NVIDIA Corp., Santa Clara, CA, USA). The model was implemented in PyTorch 2.7.1.

We train the model using the Adam optimizer with an initial learning rate of $5\times 10^{-4}$ and a MultiStep learning-rate scheduler (milestones at 50 k, 100 k, and 130 k epochs; decay factor 0.5). From the dataset generated via Physically-Stratified Sampling, 1,000,000 points are randomly selected once and used for full-batch updates at each iteration over 150,000 epochs. Full-batch training is adopted because the fixed dataset fits within the GPU memory, eliminates batch variance, and stabilizes optimization.

**Figure 5:**
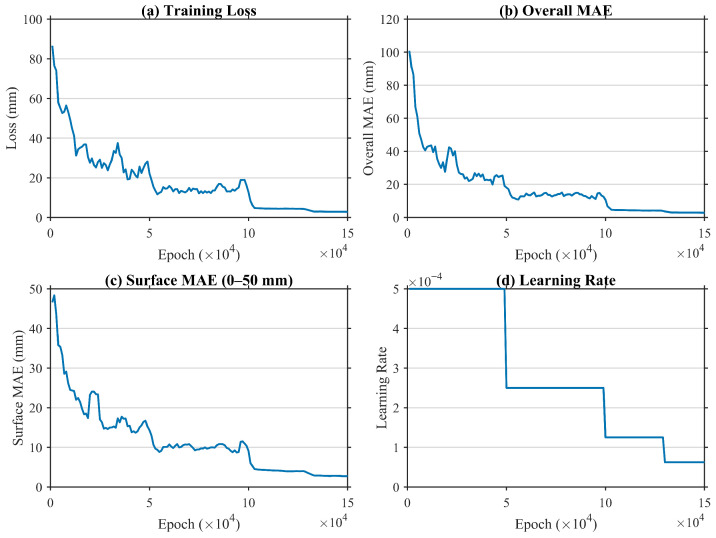
Training dynamics under physically-stratified supervision. (**a**) Total training loss; (**b**) overall MAE; (**c**) near-surface MAE (evaluated within the 50 mm Effective Critical Region); (**d**) learning-rate schedule. Stable convergence to 2–3 mm MAE is achieved after 1.5 × 10^5^ epochs.

The training dynamics are summarized in Figure 5. As shown in the figure, both the training loss and the overall MAE decrease rapidly during the early stage and gradually flatten after each learning-rate decay. The near-surface MAE within the |φ|<50 mm region follows the same trend. With the stepwise learning-rate schedule, the model converges to approximately 2–3 mm mean absolute error after $1.5\times 10^{5}$ epochs. The checkpoint with the lowest overall MAE is selected as the final model.

### 3.2. Model Evaluation and Discussion

The trained ONDP model is evaluated from four complementary aspects: prediction accuracy, generalization capability, field smoothness, and inference efficiency.

(1) Prediction Accuracy

Training convergence has been illustrated previously in Figure 5, showing that the overall mean absolute error (MAE) stably decreases below 3 mm after approximately 1.5 × 10^5^ epochs. Here, we focus on the final quantitative accuracy of the converged model.

**Figure 6:**
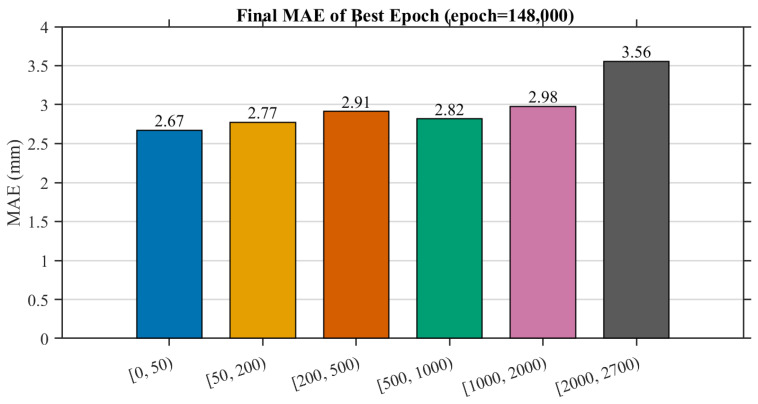
Final MAE distribution across different distance ranges at the best epoch. Near-surface zones (|φ|<50 mm) achieve the highest accuracy with an average error of 2.67 mm.

Figure 6 summarizes the bin-wise MAE distribution at the best checkpoint. Near-surface regions (|φ|<50 mm) show the lowest reconstruction error, averaging 2.67 mm, while more distant regions exhibit slightly higher deviations due to reduced sampling density. Overall, the model achieves sub-centimeter distance accuracy throughout the entire tokamak cavity, demonstrating that a pure MAE-based optimization under physically-stratified supervision is sufficient to meet the rigorous accuracy requirements of the reactor environment.

(2) Generalization Capabilities

**Figure 7:**
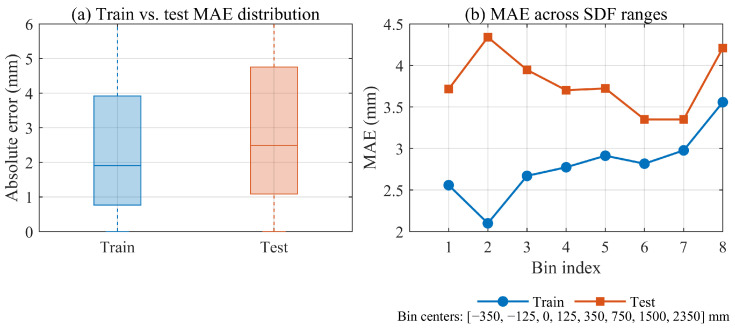
Quantitative evaluation of generalization capability. (**a**) Boxplot of absolute errors on training set (1 × 10^6^ samples) vs. unseen test set (3 × 10^5^ samples). The comparable error magnitudes confirm robust interpolation. (**b**) MAE across different spatial bins. Although the test error (orange) is higher, the maximum deviation (4.34 mm) remains safely within the 20 mm rigid margin.

To strictly validate the network’s ability to interpolate unseen geometries, we employed a hold-out validation strategy using a massive independent dataset. From the total generated pool of approximately $3\times 10^{6}$ samples, we constructed the training set by randomly sampling a subset of $1\times 10^{6}$ points. The remaining ∼$2\times 10^{6}$ samples were strictly excluded from the optimization process. For evaluation, we constructed a test set by randomly sampling $3\times 10^{5}$ points from this unseen data pool. The quantitative evaluation is presented in Figure 7.

Figure 7a compares the distribution of absolute errors between the training set and the unseen test set. The evaluation yields an overall Mean Absolute Error (MAE) of 2.80 mm on the training set and 3.69 mm on the test set. The close proximity of these error metrics demonstrates that the model effectively learned the continuous SDF manifold from the $1\times 10^{6}$ subset without overfitting to the specific training points.

Figure 7b further breaks down the MAE across different SDF distance bins. The test set MAE (orange line) follows the same spatial trend as the training set (blue line). Although a generalization gap exists, the maximum observed MAE on any unseen spatial bin is 4.34 mm (in the near-wall region). Crucially, this worst-case interpolation error remains significantly below the Rigid Safety Margin of 20 mm (which excludes the FEA-based deformation buffer). This implies that the prediction uncertainty is effectively absorbed by the engineering buffer (Error≪Margin), ensuring safe trajectory planning even under generalization conditions.

Regarding extrapolation, neural networks are known to be unreliable outside the training domain. Therefore, we define a valid operational domain Ω based on the vessel’s physical bounding box. During inference, any query point falling outside Ω is intercepted by a geometric boundary check, ensuring the planner never relies on unverified neural extrapolation.

(3) Field Smoothness

**Figure 8:**
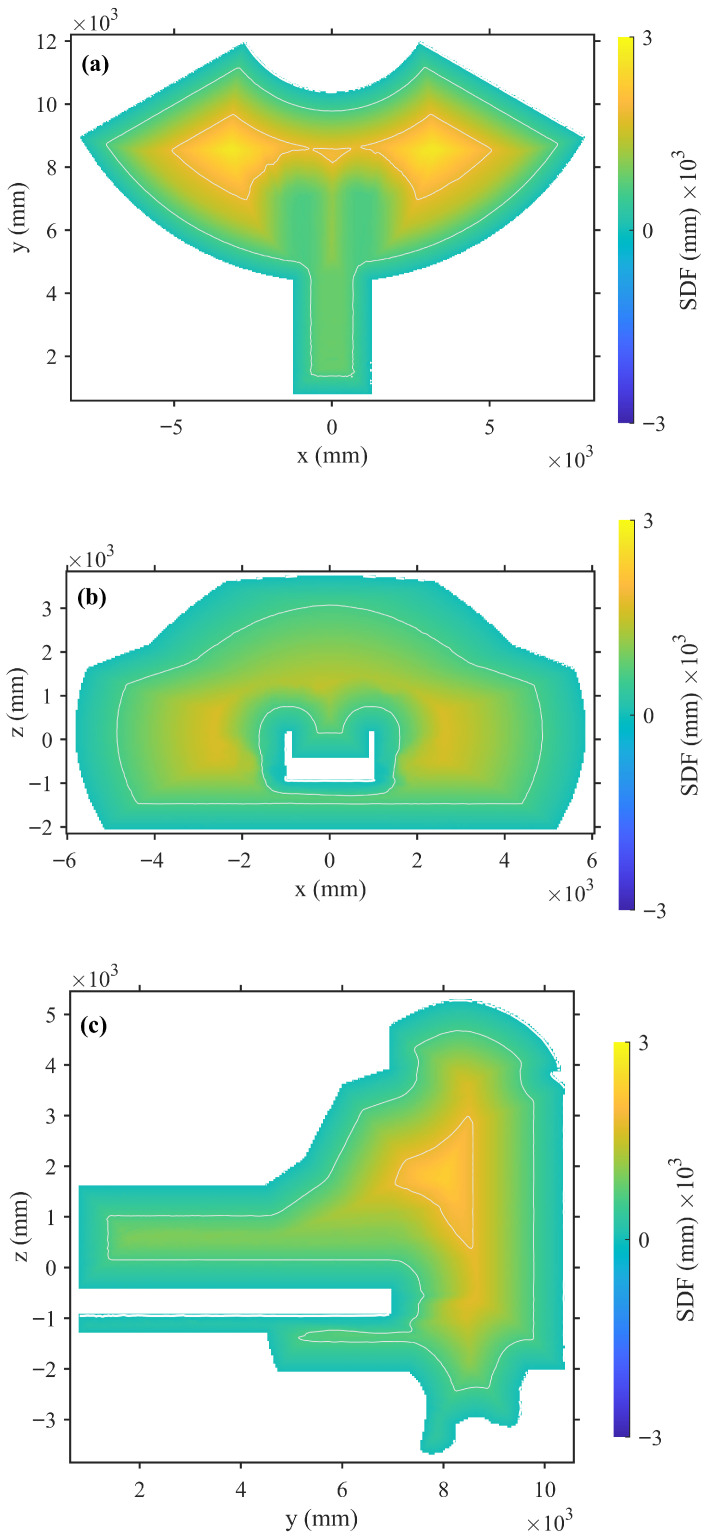
Cross-sectional visualizations of the learned ONDP on three orthogonal planes. The continuous and well-behaved transitions of the signed distance field confirm its geometric stability and smooth differentiability. (**a**) Horizontal slice (*x*–*y* plane): iso-contours are uniformly distributed, indicating smooth field transitions across the wall. (**b**) Central slice (*x*–*z* plane): the white hollow region corresponds to the internal manipulator rail channel. (**c**) Longitudinal slice (*y*–*z* plane): the rail channel region and surrounding geometry are reconstructed smoothly and consistently.

To qualitatively evaluate the spatial continuity of the learned implicit field, representative cross-sectional visualizations of the predicted ONDP are presented in Figure 8. Panels (a)–(c) correspond to orthogonal planes of the tokamak vacuum vessel (the *x*–*y*, *x*–*z*, and *y*–*z* planes, respectively). Across all sections, the signed distance field shows smooth and continuous transitions around the cavity wall, with evenly spaced iso-contours and consistent gradient orientations. No discontinuities or numerical oscillations are observed, indicating that the network successfully learns a coherent and physically meaningful implicit representation even without explicit gradient regularization.

In Figure 8b,c, the white hollow region corresponds to the manipulator rail channel located inside the vacuum vessel. This narrow and high-curvature geometry is challenging for implicit field representation, yet the ONDP accurately reconstructs both the inner and outer boundaries, demonstrating robust generalization to fine structural details. A slight smoothing effect can be noticed around extremely thin or sharp features, mainly caused by the limited sampling density and the MLP’s smoothness prior; however, this effect remains within the sub-centimeter precision required for downstream perception and motion-planning tasks.

Overall, the learned ONDP exhibits approximate Eikonal properties (∥∇φ∥≈1) across most regions, and preserves uniform sign conventions throughout the workspace. This smooth, physically accurate, and differentiable distance field provides a reliable geometric foundation for real-time collision checking, boundary-constrained trajectory optimization, and closed-loop robotic control.

(4) Inference Efficiency

**Figure 9:**
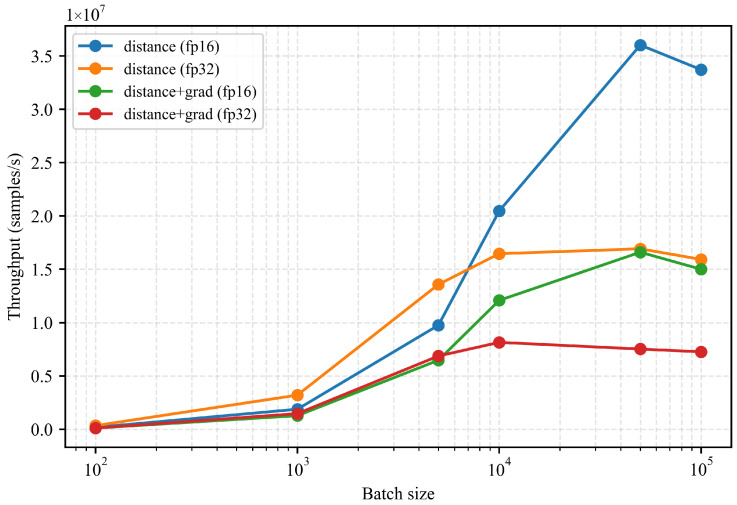
Inference throughput under different batch sizes. The ONDP achieves real-time distance and gradient queries suitable for high-frequency collision checking and optimization-based control.

We evaluate the runtime performance of the trained ONDP model on an NVIDIA RTX 4080 GPU. Thanks to its compact network architecture and continuous implicit representation, the model supports high-frequency distance and gradient queries suitable for real-time robotic deployment. Figure 9 reports the throughput under varying batch sizes. In FP16 precision, the model achieves up to ∼$3.6\times 10^{7}$ distance evaluations per second and maintains over $1.5\times 10^{7}$ evaluations per second when jointly computing both distance and gradients, enabling continuous collision checking at controller frequency.

**Figure 10:**
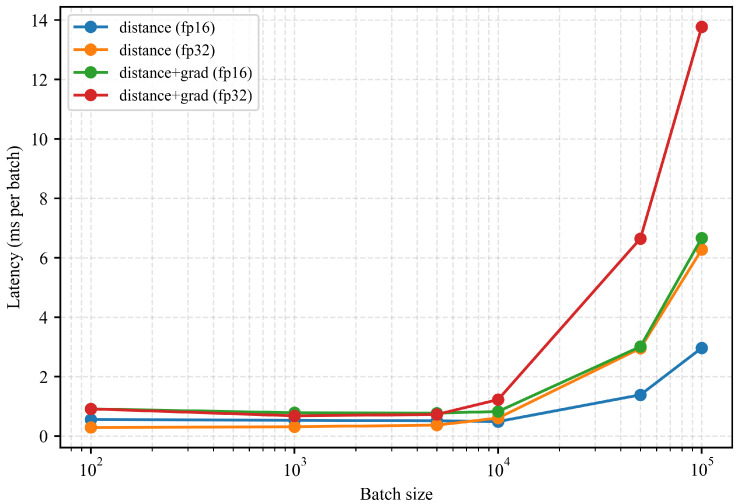
Per-batch latency under different batch sizes. Latency remains within the sub-millisecond range for practical operating conditions, supporting real-time closed-loop robotic motion planning.

Figure 10 further summarizes the per-batch latency characteristics. Across the practical batch-size range (100–10,000), the end-to-end query latency remains within a sub-millisecond scale, which is compatible with closed-loop trajectory optimization and receding-horizon motion planning. Latency increases at extremely large batch sizes due to GPU kernel scheduling and memory bandwidth saturation, but these operating points are not required for real-time execution.

The ONDP exhibits slight smoothing around extremely thin or high-curvature structures, and the extrapolation accuracy outside the sampled workspace is not guaranteed. However, within the operational region, the model preserves sub-centimeter reconstruction precision and smooth differentiability. This enables the ONDP to serve as a unified geometric module that bridges environment modeling, perception, and control in differentiable motion-planning pipelines.

### 3.3. Comparative Benchmark in Robotic Simulation

To strictly validate the feasibility of the proposed method for real-time robotic applications, we conducted a comparative benchmark focusing on query latency and simulation frequency. The experiment was performed in the high-fidelity tokamak reactor environment (186,508 triangular faces). For each simulation step, a batch of N= 10,000 points was queried to simulate a comprehensive whole-body collision check for a high-DOF robot arm. We compared our ONDP method against two standard baselines: the exact mesh-based query (accelerated by BVH) and the approximate KD-tree method.

**Figure 11:**
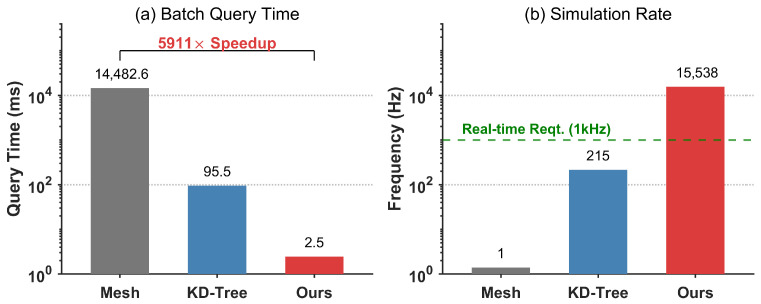
Performance benchmark comparison in a robotic simulation scenario. The experiment evaluates collision checking efficiency using a batch size of 10,000 query points against a reactor environment (186,000 faces). (**a**) Batch Query Time: The exact mesh-based method (grey) consumes 14,483 ms per batch, creating a significant computational bottleneck. In contrast, our ONDP method (red) requires only 2.45 ms, achieving a 5911× speed-up. (**b**) Simulation Frequency: The green dashed line marks the 1 kHz requirement for high-performance real-time control. While traditional methods (1.4 Hz and 215 Hz) fail to meet this standard, our method achieves approximately 15.5 kHz, ensuring high-frequency real-time responsiveness.

The quantitative results are illustrated in Figure 11. As shown in Figure 11a, the traditional mesh-based method suffers from high computational cost, requiring an average of 14,482.56 ms per batch query. This excessive latency results in a simulation update rate of only 1.4 Hz, which is prohibitive for online control tasks. Although the KD-tree method improves the query time to 95.52 ms (214.9 Hz), it still falls short of the requirements for high-dynamic interactions and suffers from discretization errors.

In strict contrast, our proposed ONDP demonstrates superior efficiency, completing the same batch query in merely 2.45 ms. This represents a 5911× speed-up compared to the exact mesh baseline. Furthermore, as highlighted in Figure 11b, our approach achieves a simulation frequency of approximately 15,538 Hz. This performance significantly exceeds the 1 kHz industry standard typically required for real-time robotic control loops. This order-of-magnitude improvement confirms that our method effectively acts as a “virtual sensor,” capable of supporting advanced control strategies where rapid collision assessment is critical.

## 4. Discussion

The proposed Occupancy-Aware Neural Distance Perception (ONDP) framework is designed to address the specific challenges of robotic maintenance in confined fusion reactors. By focusing sampling density on near-wall regions, the model achieves millimeter-level accuracy where it matters most, enabling gradient-based motion planners to generate smooth, collision-free trajectories.

### 4.1. Safety Guarantees and Operational Scope

A primary concern in deploying neural implicit representations for safety-critical tasks is the lack of absolute geometric guarantees due to approximation errors. We address operational safety through a substantial buffering strategy and a hierarchical task definition:

(1) Conservative Safety Buffer

Our evaluation indicates a high baseline reconstruction accuracy with a mean absolute error (MAE) of 2.67 mm in the critical near-wall region. Crucially, as demonstrated in the generalization analysis (Figure 7), the maximum interpolation error on completely unseen test data remains bounded below 4.5 mm (specifically 4.34 mm).

To strictly prevent collision, the planner enforces a minimum clearance constraint of $d_{\mathrm{safe}}\geq 50\;mm$. This threshold is explicitly composed of the required Rigid Safety Margin (20 mm) plus the Deformation Compensation (30 mm) derived from FEA. Therefore, even in the worst-case scenario where mechanical elasticity consumes the full 30 mm buffer, the remaining 20 mm rigid margin is still significantly larger than the maximum network error (20 mm≫4.34 mm). This confirms that the neural approximation noise is insufficient to breach the physical safety boundary.

(2) Coarse-to-Fine Manipulation Strategy

It is important to clarify that the proposed ONDP is strictly used for gross motion planning—navigating the manipulator from a start pose to a target “pre-approach” pose while maintaining the aforementioned safety distance. The method does not handle the final contact phase. Once the robot reaches the target pose, the terminal maintenance operations are delegated to a specialized end-effector. This fine manipulation stage relies on local sensing and compliance mechanisms rather than the global SDF, ensuring that the sub-centimeter accuracy of our model is fully sufficient for the navigation phase.

### 4.2. Implication for Future Motion Planning

While this study focuses on the perception and geometric representation layer, the validated inference speed establishes a necessary foundation for real-time motion planning. The differentiable nature of the proposed ONDP offers significant advantages for future trajectory optimization tasks. Unlike discrete binary collision checks or numerical gradients required by KD-trees, our model provides continuous distance values d=Φ(x) and exact analytical gradients ∇Φ(x). This allows the collision avoidance problem to be formulated as a smooth optimization constraint: $Cost\left(x\right)=\max(0,d_{\mathrm{safe}}-\Phi \left(x\right))$. With the computational bottleneck now resolved (as evidenced by the 15 kHz query frequency), future work can leverage this gradient information to inherently “push” the robot away from obstacles, combining geometric safety with robot dynamics in a multi-objective optimization framework.

### 4.3. Limitations

Despite its effectiveness, the current framework has limitations that merit discussion:

- Static Environment Assumption: The trained ONDP represents a static snapshot of the tokamak vessel and does not account for real-time changes such as movable components.
- Dependence on CAD Fidelity: The model’s accuracy is bounded by the input mesh quality. Installation tolerances or discrepancies between the CAD model and the physical reactor will propagate into the distance field.
- Offline Preprocessing Cost: To ensure high precision near vessel walls, our Physically-Stratified sampling strategy generates a large number of candidate points. While this guarantees runtime accuracy, the offline data generation process is computationally intensive compared to analytical methods.
- Extrapolation Risks: Predictions far outside the calibrated workspace are mathematically undefined. We currently mitigate this by clamping the workspace boundaries during planning.

## 5. Conclusions

This work presented an Occupancy-Aware Neural Distance Perception (ONDP) framework for accurate and continuous geometric representation of tokamak vacuum vessels.

To address the challenges of mechanical uncertainty, the proposed method introduces a Physically-Stratified Sampling strategy derived from FEA data. This approach explicitly counteracts manipulator elasticity by enforcing dense supervision in critical deformation buffers, ensuring operational safety where heuristic methods are insufficient.

By training directly in physical units, the network output strictly adheres to engineering tolerances without the need for scale recovery. Evaluation results demonstrate that the model achieves 2–3 mm near-surface accuracy and robust generalization capabilities on unseen data.

Crucially, comparative simulations validate the method’s efficiency in robotic collision-checking tasks. The proposed framework achieves a query frequency of approximately 15.5 kHz for large-scale batches, delivering a 5911× speed-up compared to traditional BVH-accelerated mesh queries. This quantitative evidence confirms that ONDP fulfills the strict real-time requirements of high-frequency robotic control loops (typically 1 kHz), overcoming the computational bottlenecks of discrete geometric methods.

Future research will focus on integrating this differentiable field into a closed-loop motion planning framework. Specifically, we aim to leverage the gradient information for torque-aware trajectory optimization, considering robot dynamics. Additionally, we plan to extend the framework to support online field updates, enabling the handling of deformable or movable components within the reactor environment.

## Figures and Tables

**Table 1:** Physically-constrained sampling intervals based on Finite Element Analysis (FEA). The critical zone is defined by the rigid margin (20 mm) plus the maximum simulated deformation (30 mm).

Layer Interval	Safety $k_{s}$	Ratio	Role & Description
[−10,0)mm	N/A	20%	Aux. Gradient Support
[0,20)mm	1.2	20%	Rigid Safety Margin (Base)
[20,50)mm	1.5	16%	Deformation Buffer (FEA)
[50,100)mm	1.0	10%	Transition Zone
[100,200)mm	1.0	8%	Planning Zone
[200,500)mm	1.0	8%	Deep Zone
[500,2700)mm	1.0	18%	Far-field Exploration

## Data Availability

The raw data supporting the conclusions of this article will be made available by the authors on request.
